# Use of Povidone-Iodine Solution for Intraoperative Antisepsis: A Systematic Review

**DOI:** 10.7759/cureus.95478

**Published:** 2025-10-26

**Authors:** Flora Petkani, Lampros Lamprogiannis, Ioannis Tsinopoulos, Nikolaos G Ziakas, Asimina Mataftsi

**Affiliations:** 1 Department of Ophthalmology, General Hospital of Florina, Florina, GRC; 2 Department of Ophthalmology, Ophthalmica Institute, Thessaloniki, GRC; 3 Second Department of Ophthalmology, Papageorgiou General Hospital/Aristotle University of Thessaloniki, Thessaloniki, GRC

**Keywords:** antisepsis, betadine, conjunctiva, ocular surgery, povidone-iodine

## Abstract

Antisepsis is a critical aspect of ocular surgery, particularly in intraocular procedures, due to the high risk of postoperative infections. Povidone-iodine is widely recognized for its broad-spectrum antimicrobial properties. This systematic review evaluates the intraoperative use of povidone-iodine applied to the conjunctiva in ocular surgeries. A systematic literature search was conducted using PubMed, Scopus, and Google Scholar for randomized controlled trials (RCTs) published between 2009 and 2023 in English that involved individuals who had undergone an ophthalmological intervention. The Preferred Reporting Items for Systematic Reviews and Meta-Analyses (PRISMA) guidelines were followed, and a PRISMA flow diagram was used to illustrate the study selection process. Keywords used included the following: ("Povidone-iodine" OR "Betadine") AND ("Conjunctiva" OR "Ocular surgery"). Studies were selected based on predefined inclusion criteria focusing on the intraoperative antiseptic application of povidone-iodine in human ocular procedures, omitting conference abstracts or unpublished studies. Four RCTs met the inclusion criteria. The evidence base is limited by the small number of included RCTs, which restricts the statistical power and generalizability of the findings. This small sample increases susceptibility to methodological bias, reduces the reliability of pooled estimates, and limits the ability to explore heterogeneity. Furthermore, the potential for publication bias cannot be excluded, particularly given the limited number of studies, which may overestimate treatment effects. These studies compared povidone-iodine 5% to other concentrations or antibiotic agents such as povidone-iodine 1%, chloramphenicol 5%, netilmicin, commercial ozonized antiseptic solution (ozone), moxifloxacin 0.5%, and gatifloxacin. The application of all agents was topically on the conjunctiva just before the ophthalmological procedure started, and only gatifloxacin was applied for three days prior to the procedure. Across all included trials, povidone-iodine demonstrated superior antiseptic efficacy, significantly reducing conjunctival bacterial flora before surgery. The comparative agents showed minimal or less consistent antimicrobial effects. No serious or minor adverse effects related to povidone-iodine use were reported in any of the trials. Povidone-iodine remains a highly effective and safe antiseptic agent for intraoperative use in ocular surgery, while a need exists for additional high-quality independently conducted RCTs to confirm the observed effects and expand the evidence base. Its broad antimicrobial spectrum and favorable safety profile support its continued and widespread application for conjunctival antisepsis, outperforming several commonly used alternatives.

## Introduction and background

The term antisepsis refers to the process of removing microorganisms that appear on the skin and mucous membranes and the simultaneous temporary reduction of normal flora through the application of topical antiseptic agents. The aim of antisepsis is to minimize the appearance of a potential infection by these microorganisms [1]. The most common aerobic microbes found to compromise the healthy state of the skin and mucous membranes, including the ocular surface, are coagulase-negative staphylococci, and *Staphylococcus epidermidis* often predominates [1]. Antiseptic agents are chemical compounds that can be used on the skin, mucous membranes, and wounds, aiming to minimize the risk of infection and sepsis [2]. Antiseptic agents are differentiated from antibiotics, which fight bacterial infection in the body, and from disinfectants, which can be used on inert surfaces and objects [1,2]. Intraoperative antisepsis is a part of a specific protocol used for the prevention of infection with pathogenic microorganisms, starting with the surgical field during the surgical operation and continuing with the postoperative treatment, as preventing postoperative infections is a matter of major importance [1,3].

Povidone-iodine, also referred to as PVP-I and Betadine, is a chemical compound widely used as an antiseptic agent, having a broad spectrum of antimicrobial activity against bacteria, fungi, protozoa, and viruses [4,5]. It is a chemical complex of the polymer povidone (polyvinylpyrrolidone) and iodine, which was synthesized in order to decrease the toxicity of iodine alone, that has been used for decades with limitations due to its irritations on the tissues [6]. This combination has the ability to release iodine in a slower motion, which leads to lower toxicity and efficient antimicrobial action [7]. It can be found in many forms, such as solution and ointment for topical usage [8]. 

Povidone-iodine is widely used in ocular surgeries as the antiseptic agent of choice. It is very crucial to be cautious with the application and the procedure in order to ensure high efficiency and patient safety [9]. The ocular surface of healthy individuals has a variety of aerobic and anaerobic bacteria, which constitute the normal flora of the conjunctiva. The most common bacteria found in the ocular surface are *Staphylococcus epidermidis* (more than 60%), non-diphtheriae *Corynebacterium* species, and *Propionibacterium acnes* [10]. Regarding the pharmacodynamics of povidone-iodine, the mechanism of action is described as the release of free iodine, which penetrates microbial cells and disrupts protein structure and synthesis, leading to cell death. Unlike antibiotics, it does not promote resistance because its action is nonspecific. Povidone-iodine exerts its antimicrobial activity primarily by targeting the cell membranes and cytoplasmic components of microorganisms. The free iodine released from povidone-iodine penetrates microbial cell walls and membranes, leading to the disruption of structural proteins and nucleic acids. This results in the denaturation of enzymes and nucleotides, which inhibits critical metabolic functions and ultimately causes microbial death. Also, povidone-iodine has an antiseptic effect, used for the disinfection of skin, mucous membrane wounds, and surgical sites, and helps in wound healing by preventing infections in cuts, burns, and abrasions [11,12]. Considering the pharmacokinetics of povidone-iodine, there is minimal systemic absorption when it is applied to intact skin and to the small conjunctival surfaces during ocular surgeries, while more absorption occurs if applied to large wounds, burns, and mucous membranes. Regarding the distribution, iodine is released slowly from the povidone complex, ensuring prolonged antimicrobial action. The systemic absorption of iodine may alter thyroid function if it is used excessively. Furthermore, absorbed iodine is metabolized in the thyroid gland and involved in thyroid hormone synthesis. It is primarily eliminated via urine, with excess iodine excreted in sweat and feces. It has plenty of therapeutic uses: in the preparation of preoperative skin, as a surgical scrub before procedures, as a wound antiseptic to prevent infections, as an oral and throat antiseptic for sore throat and dental procedures, and in the form of eye drops for eye disinfection before surgery [11,12]. Povidone-iodine is classified as an antiseptic (iodophor), widely available without a prescription, and approved by the Food and Drug Administration (FDA), World Health Organization (WHO), and other health agencies [12].

There are many advantages that support the use of povidone-iodine solution in ocular surgeries. First of all, it has a broad antimicrobial action against bacteria (Gram-positive and Gram-negative), fungi, viruses, protozoa, and even methicillin-resistant *Staphylococcus aureus *(MRSA) according to many studies. Also, it has low toxicity and is well tolerated by patients, due to the slow liberation of iodine from the povidone-iodine complex. Furthermore, chronic usage of povidone-iodine does not lead to the emergence of resistant microbes, unlike antibiotics. This is attributed to its broad-spectrum, nonspecific mode of action, which makes microbial adaptation highly unlikely. Recent studies have confirmed that microbial resistance to iodophors remains negligible even after prolonged exposure [13]. In addition, its usage and storage are easy, as it can be preserved at room temperature, and it can be easily accessible with low cost [14,15].

On the other hand, there are some disadvantages that should be considered. The absorption of iodine can negatively affect some groups of people, such as premature neonates and patients with thyroid disease, although, in the context of ophthalmic use, these systemic risks associated with intraocular antisepsis are minimal due to the shorter duration of exposure compared to burn care or neonatology [5]. Premature neonates have an underdeveloped thyroid gland and hormonal system; iodine may not be secreted appropriately, causing its accumulation in levels of toxicity [16]. It can also lead to the appearance of toxicity caused by iodine, where chronic use of povidone-iodine can cause thyroid dysfunction, hypothyroidism, or hyperthyroidism. Patients with thyroid disease, such as Graves' disease and Hashimoto's thyroiditis, are sensitive to changes in iodine absorption and availability [17]. Also, other side effects can be skin irritation, rash, or burning sensation, delayed wound healing if used excessively, and allergic reactions. Furthermore, systemic absorption includes some risks, such as metabolic acidosis and kidney dysfunction in case of excessive application, especially in burns. Interaction with lithium should be avoided, considering the risk of thyroid dysfunction [11].

The objective of this systematic review is to evaluate the intraoperative use of povidone-iodine applied to the conjunctiva in ocular surgeries, with a specific focus on its antiseptic efficacy and safety profile. By synthesizing evidence from randomized controlled trials (RCTs), this review aims to compare povidone-iodine with other antiseptic and antibiotic agents used during ophthalmological procedures and to assess its effectiveness in reducing conjunctival bacterial flora and preventing postoperative infections.

## Review

Materials and methods

This systematic review included RCTs, which were reported in the databases Google Scholar, PubMed, and Scopus from 2009 until 2023. The Preferred Reporting Items for Systematic Reviews and Meta-Analyses (PRISMA) guidelines were followed, and the PICO (Population, Intervention, Comparison, Outcome) model was used for the selection strategy of the studies, which should concern individuals who underwent an ophthalmological procedure (population), in which povidone-iodine solution was used as the antiseptic agent of choice (intervention), in comparison with another substance, for instance, an antibiotic, as the antiseptic agent (comparison), in order to define the effectiveness of povidone-iodine in antisepsis (outcome). The keywords used during the research in the databases were as follows: ("Povidone-iodine" OR "Betadine") AND ("Conjunctiva" OR "Ocular surgery"). Inclusion criteria were (a) studies which were about preoperative care of an ophthalmological procedure, (b) studies that were investigating the instillation of antiseptic agents on the conjunctiva, (c) studies which were published between 2009 and 2023, (d) studies that were comparing the effectiveness of povidone-iodine solution in different concentrations to other substances, such as antibiotics, and (e) only RCTs. Exclusion criteria were (a) studies considering postoperative antisepsis, (b) studies concerning other surgeries except ocular surgeries, (c) studies which were not in English, (d) studies including laboratory animals, and (e) non-randomized studies, observational data, or studies without direct comparators [18].

Selection of Studies, Data Collection, and Data Extraction Process

**Figure 1:**
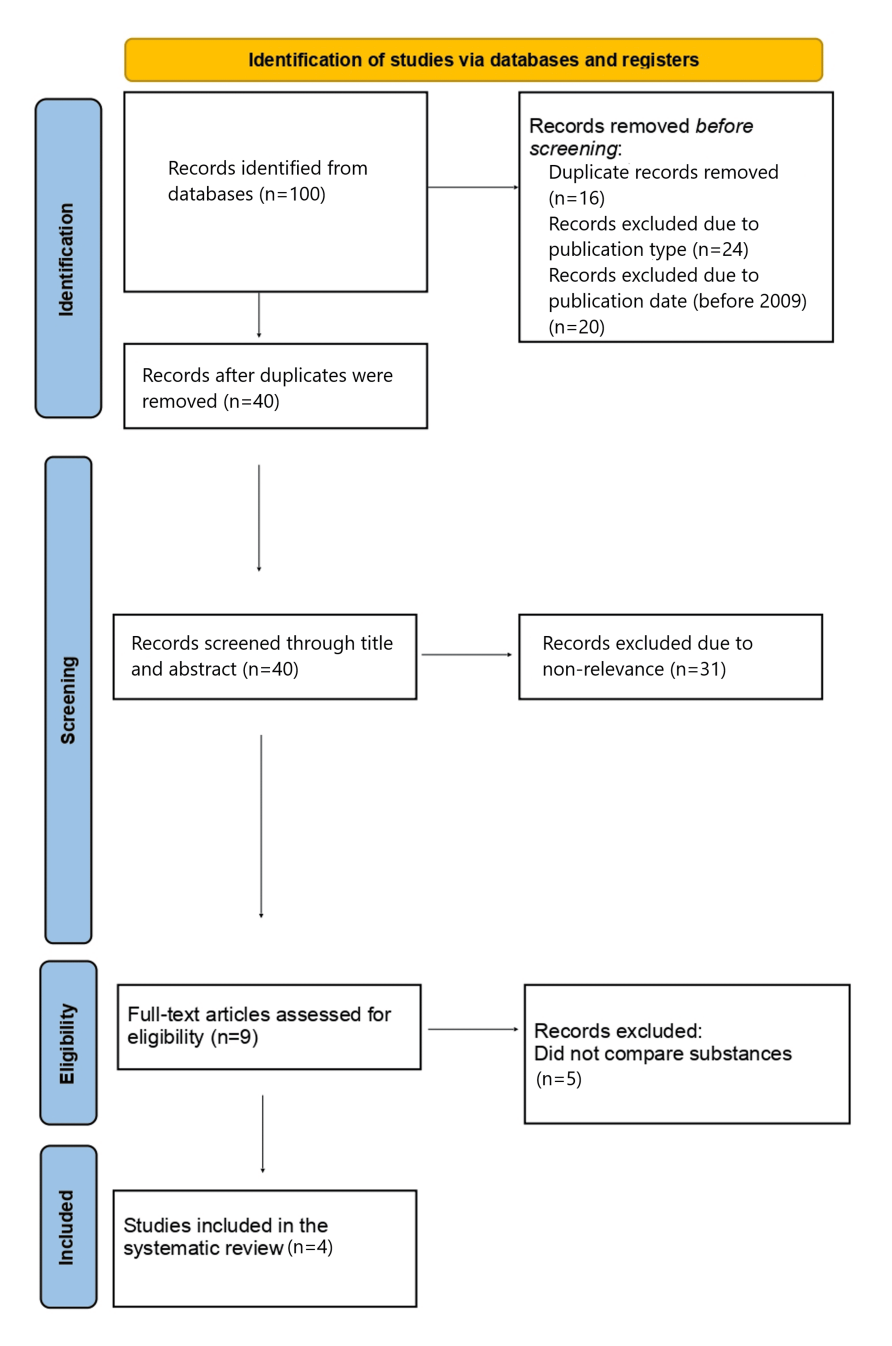
PRISMA flowchart of the included studies PRISMA: Preferred Reporting Items for Systematic Reviews and Meta-Analyses

Studies that met the inclusion criteria were selected for full-text review after being assessed, depending on their title or abstract. Two independent reviewers screened all records for inclusion. Inter-rater reliability was calculated using Cohen's kappa (κ=0.65), indicating substantial agreement. In case of disagreement, a third reviewer was consulted to reach a consensus. The search and selection strategy is shown in Figure 1.

Risk of Bias of Individual Studies

Regarding the assessment of the quality of the included studies, the Risk of Bias 2 (RoB 2) tool was used. It is a revised instrument for evaluating the risk of bias in RCTs. It was developed by Cochrane, an international organization that produces reliable and accessible health-related information. The RoB 2 tool is designed to assist researchers, reviewers, and healthcare professionals in assessing the quality and validity of RCTs, with the goal of determining whether the trial results may have been influenced by bias.

The RoB 2 tool includes five main domains for assessing the risk of bias: (1) bias arising from the randomization process, which evaluates whether randomization and allocation concealment were properly implemented to prevent predictability in assigning participants to intervention groups; (2) bias due to deviations from intended interventions, which assesses whether measures were in place to ensure that participants and personnel remained unaware of group assignments, thus avoiding performance bias; (3) bias due to missing outcome data, which evaluates whether outcome data were appropriately collected and analyzed, including how loss to follow-up and withdrawals were handled; (4) bias in measurement of the outcome, which examines whether the tools and methods used to measure outcomes were valid and reliable; and (5) bias in selection of the reported result, which assesses whether there is evidence that the reported outcomes were selectively chosen or modified after the study had begun. Each domain is rated as having either "low risk of bias", "some concerns", or "high risk of bias". The overall judgment is based on the cumulative assessment across all domains [19,20]. 

Results

Selection of Studies

The research initially gathered 100 records based on the databases PubMed, Google Scholar, and Scopus. With a more detailed reading, out of the 100 records, 16 were excluded due to duplication, 24 due to the publication type, being case reports, conference abstracts, and literature reviews, and 20 due to the date of publication, before 2009, leaving 40 studies to be considered. According to the PRISMA flowchart (Figure 1), from these 40 studies screened based on their title and abstract, only nine were selected, which were further limited to four that were included in the systematic review, because they met the inclusion criteria. The five studies that were excluded in this final stage were not acceptable because they utilized comparators that did not meet the predefined inclusion criteria, such as using alternative interventions or control conditions that were not aligned with the focus of this review.

Table 1 provides an overview of the studies included in the systematic review. The methodological heterogeneity across the included RCTs, stemming from variations in ocular procedures, povidone-iodine concentrations, and comparator agents, limits both the comparability of the studies and the strength of pooled interpretations. These inconsistencies may impact the observed outcomes and hinder the ability to draw definitive conclusions. As a result, while the review offers important insights into the potential role of povidone-iodine in ophthalmic care, the applicability of these findings to routine clinical practice should be considered with caution, particularly when generalizing to diverse surgical settings or patient populations.

**Table 1:** Summary characteristics of the reviewed studies RCT: randomized controlled trial

Author and year	Country	RCT type	Number of patients	Type of ocular surgery	Detection	Intervention	Control	Objective: results
Tofighi et al., 2023 [21]	Iran	Double-blind	260	Cataract surgery	Culture swab taken from the conjunctiva before and after the instillation of the antiseptic agent	Povidone-iodine 5%	Povidone-iodine 1%, chloramphenicol 5%	Observed that povidone-iodine 5% and chloramphenicol 5% demonstrated significant results on reducing the colonization of bacterial flora in the conjunctiva. Result: Significant decrease of the microbial load. Povidone-iodine 5% (p=0.032) has an effective antiseptic action
Trovato Battagliola et al., 2023 [22]	Italy	Triple-blind	98	Intravitreal injections	Culture swab taken from the conjunctiva before and after the instillation of the antiseptic agent	Povidone-iodine 5%	Chloramphenicol, netilmicin, ozone	Explored whether topical antibiotic prophylaxis in patients scheduled for intravitreal injections achieves surface sterility in a greater proportion of subjects as compared to povidone-iodine alone. Result: Povidone-iodine has an effective antiseptic outcome, without the contribution of other substances. The differences between the antimicrobial agents are statistically insignificant (p>0.05) after the instillation of povidone-iodine
Halachmi-Eyal et al., 2009 [10]	Israel	RCT	464	Ocular surgery	Culture swab taken from the conjunctiva after the instillation of the antiseptic agent and just before the surgery starts	Povidone-iodine 5%	Moxifloxacin 0.5%	Evaluated the effectiveness of adding moxifloxacin 0.5% to topical povidone-iodine for the preoperative reduction of bacterial recovery from the conjunctiva. Result: Povidone-iodine results in a successful antisepsis. Instillation of the antibiotic is statistically insignificant (p>0.05)
Moss et al., 2009 [23]	USA	Single-blind	129	Intravitreal injections	Culture swab taken from the conjunctiva before and after the instillation of the povidone-iodine solution	Povidone-iodine	Gatifloxacin	Compared the efficacy of three-day topical gatifloxacin use in combination with povidone-iodine versus povidone-iodine alone. Result: Povidone-iodine is an effective antiseptic agent. After the instillation of povidone-iodine, addition of an antibiotic agent has no clinical benefit (p=0.324)

Characteristics of the Selected Studies

Based on the studies included in this systematic review, results showed that povidone-iodine solution has an effective antiseptic action during ocular surgical procedures. Tofighi et al. compared the antiseptic effect of chloramphenicol 5%, povidone-iodine 5%, and povidone-iodine 1% and concluded that only povidone-iodine 5% and chloramphenicol 5% significantly reduced the microbial load [21]. In the study of Trovato Battagliola et al., povidone-iodine 5% was proven to be more efficient compared to chloramphenicol, netilmicin, and ozone instillation on the conjunctiva for antisepsis [22]. According to the study of Halachmi-Eyal et al., povidone-iodine 5% led to a bigger amount of reduction of the microbial load on the conjunctiva, compared to the insignificant contribution of the topical antibiotic moxifloxacin [10]. The study of Moss et al. was focused on the comparison of the topical use of the antibiotic gatifloxacin to povidone-iodine 5% aiming for the maximum outcome of antisepsis. Results showed that topical instillation of povidone-iodine 5% offers the best antiseptic result. 

Risk of Bias Within Studies

Regarding the assessment and the visualization of the quality of the included studies, for which the RoB 2 tool was used, the following results emerged, as presented in Table 2. More specifically, three out of the four studies were judged to raise some concerns about bias, while one study was found to have a high risk of bias. These findings suggest that the overall certainty of the evidence is moderate to low. Therefore, the conclusions drawn from this review should be interpreted with caution, as potential biases in the included studies may affect the reliability of the findings [19,20].

**Table 2:** Risk of bias assessment of the included RCT studies RCT: randomized controlled trial

Author	D1: Randomization	D2: Deviations	D3: Missing data	D4: Measurement	D5: Reported result	Overall
Tofighi et al. [21]	Low risk	Low risk	Low risk	Low risk	High risk	High risk
Trovato Battagliola et al. [22]	Low risk	Low risk	Low risk	Low risk	Some concerns	Some concerns
Halachmi-Eyal et al. [10]	Low risk	Low risk	Low risk	Low risk	Some concerns	Some concerns
Moss et al. [23]	Low risk	Low risk	Low risk	Low risk	Some concerns	Some concerns

Discussion

The aim of this systematic review was to assess and identify the effective use of povidone-iodine solution during an ocular surgical procedure. The studies included presented a comparison of povidone-iodine solution, in 5% and 1% concentrations, with other possible antiseptic substances, as chloramphenicol and other antibiotics. The results of all studies showed that povidone-iodine 5% is the gold standard for antisepsis, which is widely used with an excellent outcome. While the available evidence supports its role, further high-quality RCTs are necessary, in order to validate and strengthen the existing data.

First of all, Tofighi et al. in their randomized control trial, "Effects of chloramphenicol, povidone-iodine 1% and 5% eye drops on the colonisation of conjunctival flora in patients undergoing cataract surgery", included 260 patients. The purpose of this study was to reduce the microbial load on the conjunctiva before the patients undergo cataract surgery. Patients were split into three different groups, in which samples were collected for a microbial culture and then chloramphenicol 5%, povidone-iodine 1%, or povidone-iodine 5% was applied on the conjunctiva. After the application of the solution, new culture samples were taken. Results showed that patients who received chloramphenicol 5% and povidone-iodine 5% had a significant reduction in their microbial load, suggesting that they can be used as antiseptic agents efficiently [21]. While this study found comparable efficacy between chloramphenicol and povidone-iodine, it is important to note that most existing literature supports the superior antimicrobial activity of povidone-iodine. Therefore, these findings highlight a potential role for chloramphenicol in antiseptic protocols, though further research is needed to confirm its consistency and comparative effectiveness.

The clinical trial of Trovato Battagliola et al., "Topical antibiotic prophylaxis before intravitreal injections: a pilot study", aimed to discover the difference between the application of topical antibiotics and povidone-iodine solution, as antiseptic agents, before intravitreal injections. The total number of patients taking part in this clinical trial was 98, who were more than 18 years old. They were separated into four different groups, which received chloramphenicol, netilmicin, commercial ozonized antiseptic solution (ozone), and no substance, respectively. After the application of the topical drops, a sample was taken from the conjunctiva of the patients, before and after the instillation of povidone-iodine 5%. Before povidone-iodine instillation, the proportion of non‑sterile conjunctival swabs was 61.1% in the chloramphenicol group, 31.3% for netilmicin, 83.3% for the ozone group, and 86.5% in controls (p<0.04). After three minutes of povidone-iodine exposure, non‑sterile rates fell to 11.1% (chloramphenicol), 12.5% (netilmicin), 15.4% (control), and 25% (ozone). Importantly, the between‑group differences after povidone-iodine were not statistically significant (p>0.05). In conclusion, these findings reinforce the idea that while topical antibiotics can reduce conjunctival bacterial load, their use may be redundant when followed by povidone-iodine, which is highly effective as a standalone antiseptic [22].

An interesting result was presented in the study of Halachmi-Eyal et al., "Preoperative topical moxifloxacin 0.5% and povidone-iodine 5.0% versus povidone-iodine 5.0% alone to reduce bacterial colonization in the conjunctival sac". They tried to ascertain the addition of a topical antibiotic to the povidone-iodine application on the conjunctiva preoperatively. In this study, 464 patients were included who were scheduled to undergo an ocular surgical procedure. The study population was randomized into two groups: one group received instillation of topical moxifloxacin eye drops combined with a povidone-iodine solution, whereas the other group was treated with povidone-iodine solution alone. Samples were collected from the conjunctiva after the instillation and just before the start of the surgical procedure. Results showed that the contribution of the topical antibiotic was insignificant, hence concluding the consideration of the use of povidone-iodine alone as appropriate and the addition of the topical antibiotic as not necessary [10]. Povidone-iodine has a rapid onset of action, while antibiotics require time to penetrate ocular tissues and achieve therapeutic concentrations, because their mechanism of action is slower, targeting specific bacterial processes like protein synthesis. Thus, timing favors antiseptics like povidone-iodine for immediate microbial kill. In addition, povidone-iodine is a broad-spectrum antiseptic with a non-selective mechanism, acting by denaturing proteins and oxidizing cellular components. This makes it fast-acting and effective against a wide range of microorganisms, in contrast to topical antibiotics that work by inhibiting bacterial DNA synthesis, which takes more time and is limited to bacteria. Furthermore, frequent use of antibiotics in the eye can lead to the selection of resistant ocular flora, while there is no known resistance mechanism in microbes against povidone-iodine [11,22].

Furthermore, Moss et al. published their study, "A prospective randomized evaluation of topical gatifloxacin on conjunctival flora in patients undergoing intravitreal injections", aiming to evaluate the combination of antibiotic gatifloxacin and povidone-iodine on conjunctival application as an antiseptic agent against the use of povidone-iodine alone. The patients included were scheduled to undergo intravitreal injections, were 129 in total, and were divided into two different groups. Only one of the groups applied the topical antibiotic for three days before the procedure, and then both groups received povidone-iodine. Results proved that despite the reduction of microbial growth that was noticed with the topical antibiotic addition, the combination of the two substances had an insignificant outcome. Due to its broad-spectrum and nonspecific antimicrobial activity, povidone-iodine likely eliminates a wide range of microbial flora. This extensive microbial clearance significantly reduces the baseline microbial burden, thereby limiting the potential for additional benefit from adjunctive antimicrobial agents. In conclusion, topical application of povidone-iodine solution has a satisfactory and efficient antiseptic outcome [23].

An important research gap is highlighted regarding the uncertainty around the optimal exposure time of the intraocular application of povidone-iodine during surgery. While many studies focus on the concentration of povidone-iodine, fewer rigorously compare different contact times or provide a strong consensus about how long the antiseptic should remain on ocular tissues to achieve the maximal reduction of bacterial load without undue toxicity. In the setting of intravitreal injections, some experts and guidelines recommend as little as 30 seconds of contact time with povidone-iodine 5% to reduce conjunctival flora significantly [24]. However, for intraocular surgery such as cataract operations, the European Society of Cataract and Refractive Surgeons (ESCRS) and other bodies often recommend three minutes of exposure to 5-10% povidone-iodine on the conjunctiva, cornea, lid margins, and periocular skin [25]. The variation in these recommendations and in the durations used in published studies suggests that the exposure time may influence outcomes significantly. It remains possible that shorter times may be sufficient under some conditions (e.g., in low bacterial load or when using particular povidone-iodine concentrations), while longer contact may improve antisepsis but at the risk of higher tissue irritation or toxicity. In light of this, future research should include systematic investigations of exposure time, which compare both antiseptic efficacy (bacterial cultures, rates of endophthalmitis) and safety (ocular surface toxicity, endothelial/retinal effects).

## Conclusions

The use of povidone-iodine as an antiseptic agent in surgical procedures is an important issue that has been studied and analyzed by many researchers because of its major importance. There are many studies supporting its usage, highlighting its advantages, such as the efficient antiseptic activity, well-tolerated ability by humans, and its low cost and easy access. Although limited in number, the included studies show generally consistent findings; however, given the moderate risk of bias, the heterogeneity in patient populations and surgical procedures, and the small number of RCTs, the overall certainty of evidence should be considered moderate to low. Furthermore, many studies focus on the discovery of the most appropriate concentration of the povidone-iodine solution, with 5% being the concentration of choice, followed by other possible concentrations that can be safely and satisfactorily used. These findings highlight the need for additional research to identify the optimal contact time of the solution on human tissue to achieve maximal antiseptic effectiveness, as well as to explore comparative and synergistic effects between povidone-iodine and other antiseptic agents. An issue of great importance is to differentiate the specific needs of each type of surgical procedure and the possible complications, in order to be able to react properly and possibly prevent them.
